# Network pharmacology based research into the effect and mechanism of Yinchenhao Decoction against Cholangiocarcinoma

**DOI:** 10.1186/s13020-021-00423-4

**Published:** 2021-01-21

**Authors:** Zhiqiang Chen, Tong Lin, Xiaozhong Liao, Zeyun Li, Ruiting Lin, Xiangjun Qi, Guoming Chen, Lingling Sun, Lizhu Lin

**Affiliations:** 1 The First School of Clinical Medical Sciences, Guangzhou University of Chinese Medicine, 510405 Guangzhou, China; 2 Department of Oncology, The First Affiliated Hospital of Guangzhou University of Chinese Medicine, No. 16, Jichang Road, Baiyun District, 510405 Guangzhou, China

**Keywords:** Yinchenhao decoction, Network pharmacology, Mechanism, Cholangiocarcinoma

## Abstract

**Background:**

Cholangiocarcinoma refers to an epithelial cell malignancy with poor prognosis. Yinchenhao decoction (YCHD) showed positive effects on cancers, and associations between YCHD and cholangiocarcinoma remain unclear. This study aimed to screen out the effective active components of Yinchenhao decoction (YCHD) using network pharmacology, estimate their potential targets, screen out the pathways, as well as delve into the potential mechanisms on treating cholangiocarcinoma.

**Methods:**

By the traditional Chinese medicine system pharmacology database and analysis platform (TCMSP) as well as literature review, the major active components and their corresponding targets were estimated and screened out. Using the software Cytoscape 3.6.0, a visual network was established using the active components of YCHD and the targets of cholangiocarcinoma. Based on STRING online database, the protein interaction network of vital targets was built and analyzed. With the Database for Annotation, Visualization, and Integrated Discovery (DAVID) server, the gene ontology (GO) biological processes and the Kyoto encyclopedia of genes and genomes (KEGG) signaling pathways of the targets enrichment were performed. The AutoDock Vina was used to perform molecular docking and calculate the binding affinity. The PyMOL software was utilized to visualize the docking results of active compounds and protein targets. In vivo experiment, the IC_50_ values and apoptosis rate in PI-A cells were detected using CCK-8 kit and Cell Cycle Detection Kit. The predicted targets were verified by the real-time PCR and western blot methods.

**Results:**

32 effective active components with anti-tumor effects of YCHD were sifted in total, covering 209 targets, 96 of which were associated with cancer. Quercetin, kaempferol, beta-sitosterol, isorhamnetin, and stigmasterol were identified as the vital active compounds, and AKT1, IL6, MAPK1, TP53 as well as VEGFA were considered as the major targets. The molecular docking revealed that these active compounds and targets showed good binding interactions. These 96 putative targets exerted therapeutic effects on cancer by regulating signaling pathways (e.g., hepatitis B, the MAPK signaling pathway, the PI3K-Akt signaling pathway, and MicroRNAs in cancer). Our in vivo experimental results confirmed that YCHD showed therapeutic effects on cholangiocarcinoma by decreasing IC_50_ values, down-regulating apoptosis rate of cholangiocarcinoma cells, and lowering protein expressions.

**Conclusions:**

As predicted by network pharmacology strategy and validated by the experimental results, YCHD exerts anti-tumor effectsthrough multiple components, targets, and pathways, thereby providing novel ideas and clues for the development of preparations and the treatment of cholangiocarcinoma.

## Background

Cholangiocarcinoma refers to an uncommon biliary adenocarcinoma originating from epithelial cells of the biliary tract. It falls into two subtypes based on anatomical location, namely, intrahepatic cholangiocarcinoma and extrahepatic cholangiocarcinoma [1]. The morbidity and mortality of intrahepatic cholangiocarcinoma have been rising in recent years, which is not consistent with extrahepatic cholangiocarcinoma [2, 3].

The occurrence of cholangiocarcinoma is likely to be associated with a range of risk factors (e.g., cirrhosis, hepatitis B and C infection, obesity, as well as diabetes mellitus), yet the etiology remains unclear [4–6]. Existing treatments for cholangiocarcinoma consist of surgery, chemotherapy and targeted radiation. For early stage cholangiocarcinoma patients, surgical treatment is most commonly required for tumor resection, thereby affecting patients’ postoperative recovery with the five-year survival rate (20–40%), and the surgery is not recommended for patients with advanced cholangiocarcinoma [7, 8]. Currently, Regimens based on 5-fluorouracil have been proved to slightly prolong survival time, and cisplatin combined with gemcitabine treatments have achieved the similarresults in comparison with gemcitabine alone [9, 10],whereaswestern medicineis likely to cause adverse reactions [11]. Thus, traditional Chinese medicine, i.e., a complementary and alternative approach, is considered in the treatment of cholangiocarcinoma.

Yinchenhao decoction (YCHD) consists of three herbs, namely, *Herba Artemisiae Scopariae* (Yinchenhao, YCH), *Gardeniae Fructus* (Zhizi, ZZ) and *Radix Rhei et Rhizoma* (Dahuang, DH). YCHD is closely correlated with liver and gallbladderin accordance with the traditional Chinese medicine theory, which can eliminate dampness and heat of liver and gallbladder. Clinically, it has been widely adopted to treat cholestasis, liver disorders and metabolic diseases [12, 13]. Previous researches have also reported that YCHD had positive effects on various cancers including pancreatic cancer and ascites hepatoma [14, 15], however, the associations between YCHD and cholangiocarcinoma haven’t been thoroughly studied and require further researches. Covering numerous chemical compounds acting on multiple targets, the mechanisms of YCHD remain unclear. Network pharmacology refers to a promising methodology integrating pharmacology, molecular biology, electronic technology and bioinformatics to form network relationship among active ingredients of Chinese formulas, relevant targets, pathways, as well as diseases [16].

**Fig. 1:**
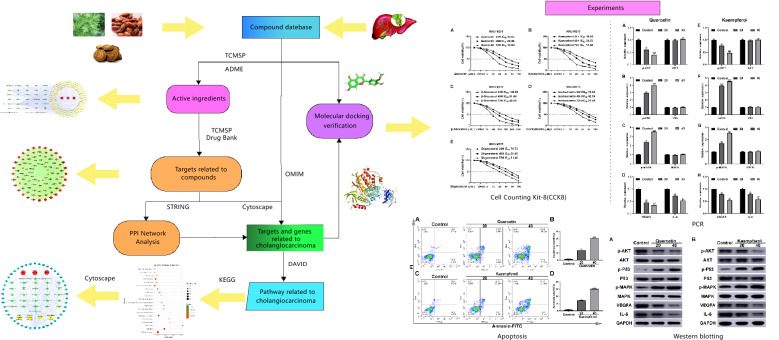
The whole framework of this study based on network pharmacology for investigating pharmacological mechanisms of YCHD acting on cholangiocarcinoma

The present study was designed to delve into the mechanisms of YCHD on cholangiocarcinoma using network pharmacology methods, as an attempt to be referenced for subsequent pharmacological studies and clinical treatments of cholangiocarcinoma.The flowchart of YCHD in treating cholangiocarcinoma was provided in Fig. 1.

## Materials and Methods

### Constructing database of candidate compounds

All constituents of YCHD were achieved from the Traditional Chinese Medicine Systems Pharmacology Database and Analysis Platform (http://lsp.nwu.edu.cn/tcmsp.php, TCMSP), Traditional Chinese Medicines Integrated Database (http://119.3.41.228:8000/tcmid/, TCMID), and Traditional Chinese Medicine Database@Taiwan (http://tcm.cmu.edu.tw/) [17–19]. Oral bioavailability (OB) and drug-likeness (DL) indices recommended by TCMSP were employed to verify druggability of each candidate. OB was referred to as the extent and rate to which the active drug ingredient or active moiety from the drug product was absorbed and available at the site of drug action [20]. High OB appeared to be more probably a drug-like ingredient. DL index was adopted to assess whether the compounds were chemically suitable [21]. Compounds that overcome both OB and drug-like phase screening would be recognized as candidate compounds more possibly. As TCMSP suggests, the molecules with OB ≥ 30% and DL ≥ 0.18 were preserved to display relatively better pharmacologically and then screened out as candidate compounds for subsequent analysis. As a result, thirty-two compounds of YCHD were identified.

### Mining cholangiocarcinoma associated targets and target-genes

Protein targets associated with cholangiocarcinoma were provided by the GeneCard databases with “cholangiocarcinoma” as the keywords. All the targets were only limited to “homo sapiens”. Subsequently, protein names of all targets were switched to corresponding gene names in the website of UniProt (https://www.uniprot.org/) or PharmMapper (http://lilab.ecust.edu.cn/pharmmapper/). Furthermore, the same procedure was also performed to extract relevant target genes of herbs of YCHD.

### Conducting PPI network

Given that the search of protein interactions and the interaction network is the critical procedure for gaining the insights into cellular organization, bioprocess, and functions, associated targets were input into STRING (Version 10.5, https://string-db.org/) to delve into protein-protein interactions. The network nodes and edges denote proteins and protein-protein associations, respectively. Two PPI interactive networks were built and then visualized by Cytoscape software (version 3.6.0), including estimated YCHD components and cholangiocarcinoma related targets. After merging these two networks as a candidate network following the intersection of PPI data, topological features were analyzed to sift a core PPI network.

### Gene Ontology (GO) and pathway enrichment analysis

The DAVID web server (Functional Annotation Result Summary, https://david.ncifcrf.gov/summary.jsp) was adopted to conduct GO enrichment analysis for the candidate target protein obtained after network merging. Subsequently, Kyoto Encyclopedia of Genes and Genomes (KEGG) pathway enrichment analysis was conducted to explore biological pathways where relevant proteins were covered. A P value ≤ 0.05 was considered significant, and enriched GO terms were identified by the hypergeometric test. A bubble chart was plotted via the OmicShare platform, a free online platform to conduct data analysis (http://www.omicshare.com/tools).

### Validation of compound‐target interaction

The crystal structures of hub protein targets were obtained from the Protein Data Bank (PDB, https://www.rcsb.org/). The three-dimensional structures of candidate active compounds were downloaded from the PubChem (https://pubchem.ncbi.nlm.nih.gov/), an open archive for chemical information. The downloaded active compounds and hub protein targets were converted to the pdbqt format via the AutoDockTools (version 1.5.6, http://autodock.scripps.edu/). The AutoDock Vina (http://vina.scripps.edu/) was used to perform molecular docking and calculate the binding affinity. The docking results of active compounds and protein targets were visualized with PyMOL software (version 2.2, https://pymol.org/2/).

### Cell cultures and cell viability measurements

CCK-8 kit was used to evaluate the effects of quercetin, kaempferol, beta-sitosterol, isorhamnetin, and stigmasterol on the KKU-M213 cell lines. The KKU-M213 cell lines seeded into 96-wall plates at a density of 8 × 10^3^ cell/100 ul were treated with various concentrations of quercetin, kaempferol, beta-sitosterol, isorhamnetin, and stigmasterol for 24, 48, and 72 hours, respectively. Then the cells were incubated with 10 µl CCK-8 solution for 90 min. A microculture plate reader was used to measure the optical density at 450 nm. The SPSS 20.0 software was adopted to calculate 50% inhibitory concentration (IC50) value.

### Apoptosis detection

The PI-A cells in logarithmic growth of each group containing quercetin and kaempferol treatment were collected and seeded into 96-wall plates at a density of 1 × 10^6^ cell/2 m, and incubated overnight at 37 °C with 5% CO_2_. The treated cells were washed with cold PBS solution, incubated with Annexin-FITC, and then placed in the water bath for 30 min. The Cell Cycle Detection Kit was applied to detect the cell circle.

### Real‐time PCR assay

After an appropriate amount of cells in each group were collected, the supernatant was decanted and then washed with PBS. The TRIzol solution was added to extract total RNA of cells. The ultraviolet spectrophotometer was used to assess the RNA purity and concentration. AKT, P53, MAPK and VEGFA mRNA expression reversed transcription to cDNA. The above cDNA was used as template and amplified according to the real-time PCR instruction. The 20 µL PCR reaction system included 10 µL SYBR Green Mix, 1 µL each for upstream and downstream primer sequences, 2 µL template cDNA, and 2 µL DNase and RNase-free water. They were pre-denaturated at 94 °C for 5 min, denaturated at 94 °C for 30 s, annealed at 60 °C for 30 s, extended at 72 °C for 30 s, totally circulating 40 times. The β-actin acting as internal reference, the relative expression of each target mRNA was calculated by ABI Prism® SDS 2.0.3 using the 2^−ΔΔCt ^method.

### Western blot assay

The cells of each group were lysed with RIPA lysate for 30 min, and then transferred to the centrifuge tube. After centrifuging at 12,000 r/min for 10 min, the supernatant was extracted. The quantitative protein concentration was detected by BCA Protein Assay Kit. After separated by SDS-PAGE, 50 µg protein samples were transferred onto the PVDF membrane, sealed with 5% skim milk powder at room temperature for 1 h, and then washed with PBST solution. Rabbit anti AKT, P53, MAPK, VEGFA and IL-6 monoclonal antibodies (1:1000) were added respectively to incubate overnight at 4 °C, then the membrane was washed again, the corresponding secondary antibody were added, and the ECL kit was used to stain. The gray value of each imaging protein band was analyzed by the Gel Imaging System, and compare the relative expression change of each group protein with β-actin as internal reference.

### Statistical analysis

The data were expressed as mean ± SD. The results were analyzed using GraphPad Prism 6.02 and SPSS 20.0 software. Student’s t-tests were developed to compare the between-group quantitative data, and p < 0.05 revealed significant difference.

## Results

### Compound-compound target network analysis

**Fig. 2:**
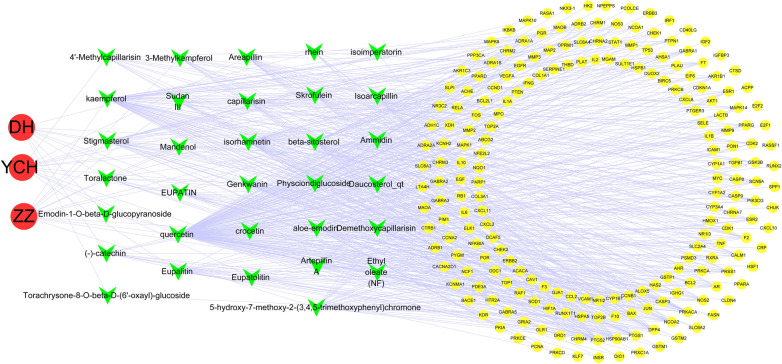
The compound-compound target network for YCHD on treating cholangiocarcinoma

32 active compounds were screened out in total following the OB ≥ 30% and DL ≥ 0.18 criteria. Table 1 lists the 32 active compounds. With the top 5 degrees, quercetin, kaempferol, beta-sitosterol, isorhamnetin, and stigmasterol were considered as the vital active compounds. Figure 2 shows that the compound-compound target network consisted of 244 nodes (namely, 3 herbs, 32 active compounds and 209 compound targets) and 640 edges. According to this network, targets in the interior circle exhibited more interactions than those in the exterior circle, and considerable targets were regulated by multiple compounds. For instance, Prostaglandin G/H synthase 2 was modulated by a range of compounds (e.g., crocetin, ammidin and isorhamnetin). It is therefore speculated that active compounds of YCHD might impact multiple targets to effectively treat cholangiocarcinoma. In this network, relationships between active compounds and compound targets as well as potential pharmacological effects of YCHD were visually illustrated.

**Table 1:** Information for candidate active compounds from YC, ZZ, DH herbs

ID	Molecule name	OB (%)	HL	Herbs	CAS number
MOL000354	Isorhamnetin	49.6	0.31	YC	480-19-3
MOL000358	Beta-sitosterol	36.91	0.75	YC/ZZ/DH	83-46-5
MOL004609	Areapillin	48.96	0.41	YC	83162-82-7
MOL005573	Genkwanin	37.13	0.24	YC	437-64-9
MOL007274	Skrofulein	30.35	0.3	YC	6601-62-3
MOL008039	Isoarcapillin	57.4	0.41	YC	85819-50-7
MOL008040	Eupalitin	46.11	0.33	YC	29536-41-2
MOL008041	Eupatolitin	42.55	0.37	YC	29536-44-5
MOL008043	Capillarisin	57.56	0.31	YC	56365-38-9
MOL008045	4’-Methylcapillarisin	72.18	0.35	YC	520-12-7
MOL008046	Demethoxycapillarisin	52.33	0.25	YC	61854-36-2
MOL008047	Artepillin A	68.32	0.24	YC	N/A
MOL000098	Quercetin	46.43	0.28	YC/ZZ	73123-10-1
MOL001406	Crocetin	35.3	0.26	ZZ	27876-94-4
MOL001941	Ammidin	34.55	0.22	ZZ	482-44-0
MOL004561	Sudan III	84.07	0.59	ZZ	85-86-9
MOL000422	Kaempferol	41.88	0.24	ZZ	520-18-3
MOL000449	Stigmasterol	43.83	0.76	ZZ	83-48-7
MOL001494	Mandenol	42	0.19	ZZ	544-35-4
MOL001942	Isoimperatorin	45.46	0.23	ZZ	482-45-1
MOL002883	Ethyl oleate (NF)	32.4	0.19	ZZ	111-62-6
MOL003095	5-Hhydroxy-7-methoxy-2-(3,4,5-trimethoxyphenyl)chromone	51.96	0.41	ZZ	18103-41-8
MOL007245	3-Methylkempferol	60.16	0.26	ZZ	1592-70-7
MOL002235	EUPATIN	50.8	0.41	DH	19587-65-6
MOL002259	Physciondiglucoside	41.65	0.63	DH	84268-38-2
MOL002268	Rhein	47.07	0.28	DH	478-43-3
MOL002280	Torachrysone-8-O-beta-D-(6’-oxayl)-glucoside	43.02	0.74	DH	N/A
MOL002281	Toralactone	46.46	0.24	DH	41743-74-2
MOL002288	Emodin-1-O-beta-D-glucopyranoside	44.81	0.8	DH	23313-21-5
MOL002297	Daucosterol_qt	35.89	0.7	DH	474-58-8
MOL000471	Aloe-emodin	83.38	0.24	DH	481-72-1
MOL000096	(−)-catechin	49.68	0.24	DH	154-23-4

*YC* Yinchenhao (Artemisiae Scopariae Herba), *ZZ* Zhizi (Gardeniae Fructus), *DH* Dahuang (Radix Rhei et Rhizoma)

### Identification of intersection target genes

**Fig. 3:**
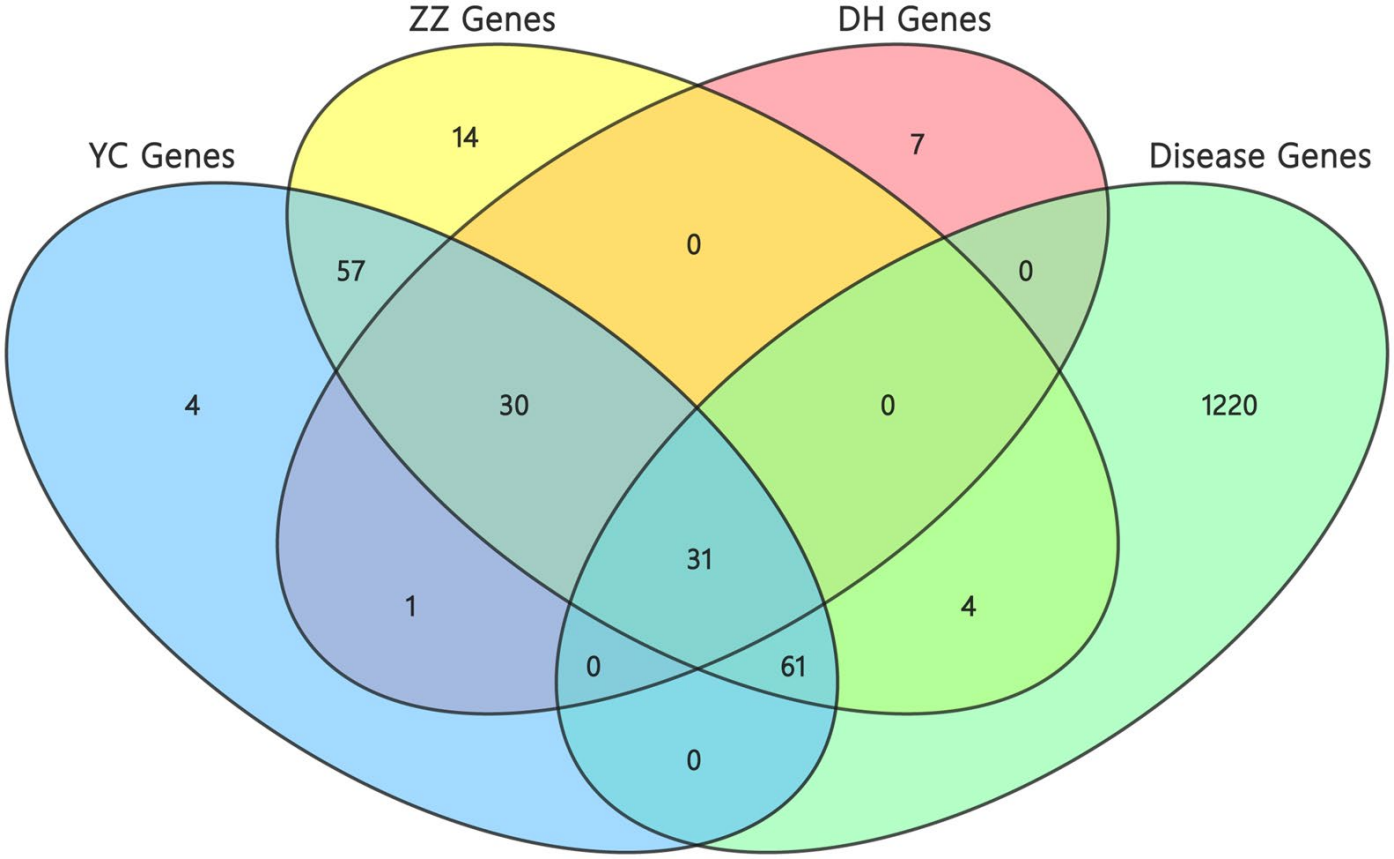
The venn diagram of 96 target genes from YCH, ZZ, DH herbs

The network scores of genes were mapped from the mentioned calculation to the targets of cholangiocarcinoma disease genes, as well as target genes of herbs of YCHD, respectively, expressing the relationship between the three herbs of YCHD and cholangiocarcinoma disease. 96 target genes were identified affected by cholangiocarcinoma and regulated by YCH, ZZ and DH (Fig. 3; Table 2). As revealed from the results, ZZ were more critical to treat cholangiocarcinoma.

**Table 2:** Information for candidate targets from compounds of YC, ZZ, DH herbs

No.	Target	Uniprot ID*	No.	Target	Uniprot ID*	No.	Target	Uniprot ID*	No.	Target	Uniprot ID*
1	NOS2	P35228	25	TGFB1	P01137	49	HIF1A	Q16665	73	GSTP1	P09211
2	PTGS1	P23219	26	PON1	P27169	50	HSPA5	P11021	74	NFE2L2	Q16236
3	ESR1	P03372	27	F10	P00742	51	ERBB2	P04626	75	NQO1	P15559
4	PTGS2	P35354	28	RXRA	P19793	52	PPARG	P37231	76	PARP1	P09874
5	MAPK14	Q16539	29	MMP9	P14780	53	HMOX1	P09601	77	AHR	P35869
6	GSK3B	P49841	30	MMP3	P08254	54	CYP1A2	P05177	78	NR1I3	Q14994
7	PIK3CG	P48736	31	EGFR	P00533	55	MYC	P01106	79	CHEK2	O96017
8	PRSS1	P07477	32	AKT1	P31749	56	CYP1A1	P04798	80	CLDN4	O14493
9	CCNA2	P20248	33	VEGFA	P15692	57	IL1B	P01584	81	PPARA	Q07869
10	CALM1	P0DP23	34	CCND1	P24385	58	SELE	P16581	82	PPARD	Q03181
11	CHEK1	O14757	35	BCL2L1	Q07817	59	PTGER3	P43115	83	HSF1	Q00613
12	F7	P08709	36	CDKN1A	P38936	60	CXCL8	P10145	84	CRP	P02741
13	F2	P00734	37	PLAU	P00749	61	BIRC5	O15392	85	SPP1	P10451
14	NOS3	P29474	38	MMP2	P08253	62	HSPB1	P04792	86	RASSF1	Q9NS23
15	PGR	P06401	39	MAPK1	P28482	63	IL2	P60568	87	E2F1	Q01094
16	CHRM3	P20309	40	IL10	P22301	64	NR1I2	O75469	88	CD40LG	P29965
17	GABRA5	P31644	41	EGF	P01133	65	CCNB1	P14635	89	IRF1	P10914
18	BCL2	P10415	42	RB1	P06400	66	THBD	P07204	90	ERBB3	P21860
19	BAX	Q07812	43	TNF	P01375	67	COL1A1	P02452	91	HK2	P52789
20	CASP9	P55211	44	IL6	P05231	68	IFNG	P01579	92	GSTM1	P09488
21	JUN	P05412	45	TP53	P04637	69	ALOX5	P09917	93	MAPK8	P45983
22	CASP3	P42574	46	ELK1	P19419	70	PTEN	P60484	94	SLPI	P03973
23	CASP8	Q14790	47	RAF1	P04049	71	MPO	P05164	95	ADH1C	P00326
24	PRKCA	P17252	48	MMP1	P03956	72	ABCG2	Q9UNQ0	96	MAOA	P21397

### Compound‐compound target‐cholangiocarcinoma Target-Other Human Proteins’ PPI Network analysis

**Fig. 4:**
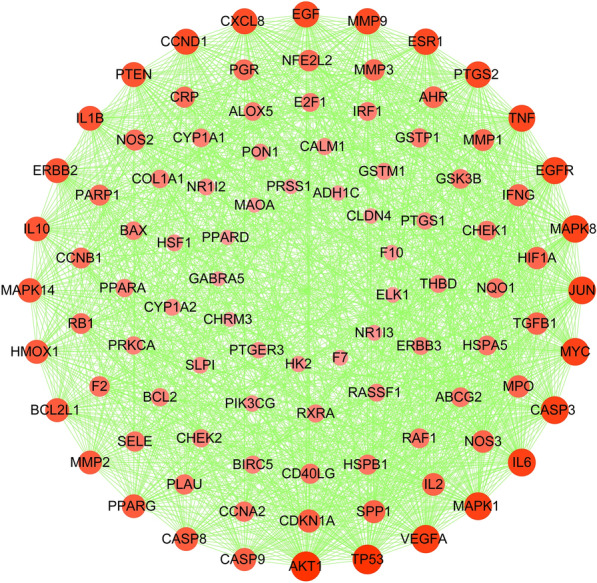
The Disease targets-PPI network

To identify the most highly connected nodes from others, the compound-compound target-cholangiocarcinoma target-other human proteins’ PPI network analysis was conducted. This network is illustrated in Fig. 4, covering 96 nodes and 1496 edges. YCHD exerted its therapeutic effects on cholangiocarcinoma through multiple protein targets. The nodes with top 5 degrees, including AKT1, IL6, MAPK1, TP53, and VEGFA, refer to the major targets in treating cholangiocarcinoma.

### GO and pathway enrichment analysis

**Fig. 5:**
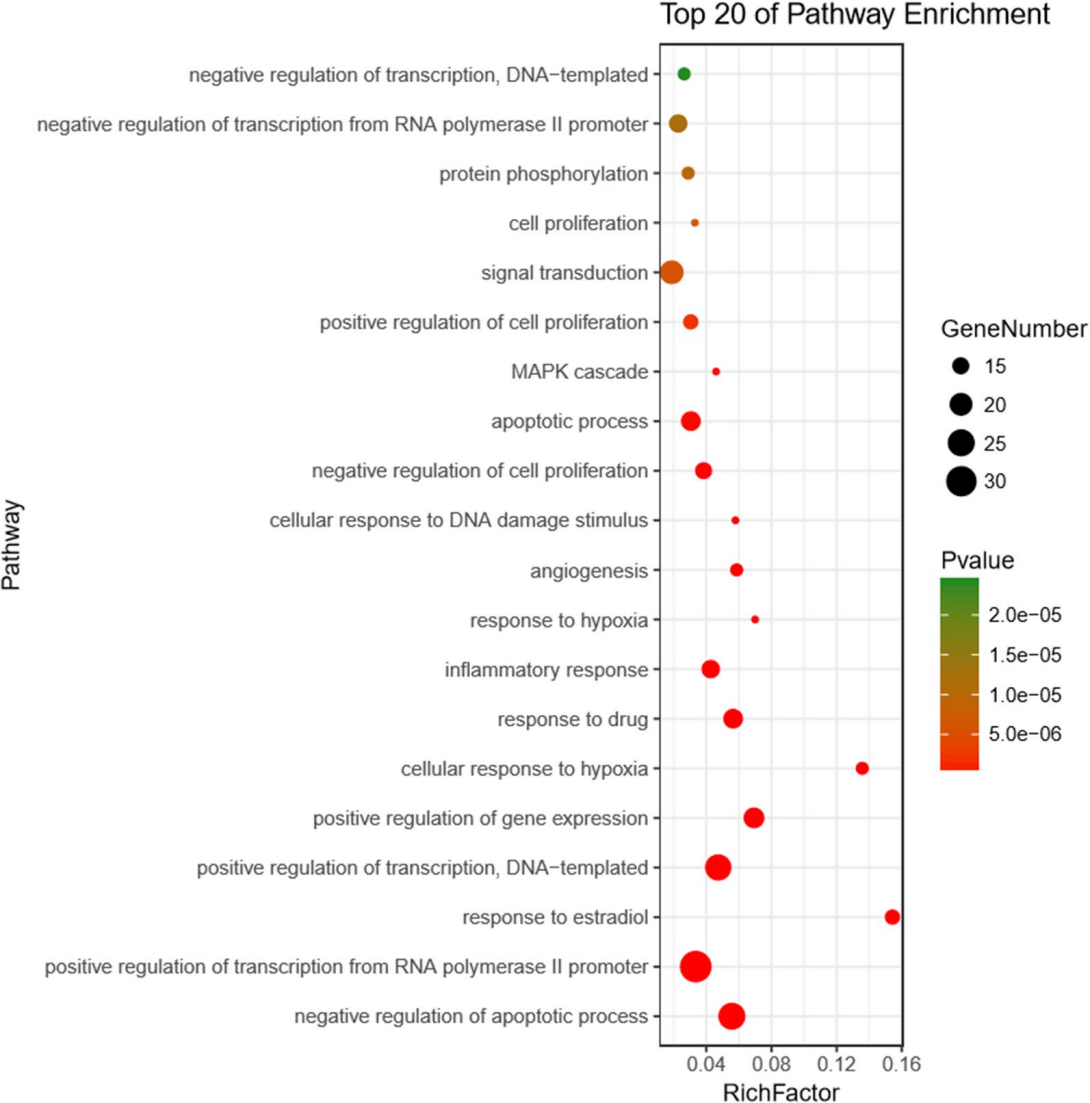
GO analysis for the major targets of YCHD

To clarify the function of estimated protein targets, the GO biological process enrichment analysis was conducted. The top 20 noticeably enriched GO terms are listed in Fig. 5; Table 3. As suggested from the results, the targets of YCHD displayed tight relations to the major biological process, which included positive regulation of transcription from RNA polymerase II promoter, negative regulation of apoptosis process, and positive regulation of transcription, DNA-templated. Thus, compound targets of YCHD exhibited similar functions to the corresponding genes of cholangiocarcinoma disease.

**Table 3:** Main GO pathways significantly relating to major hubs

Term	Pathway	Count	Pop hits	P Value
GO:0045944	Positive regulation of transcription from RNA polymerase II promoter	32	981	9.79E−16
GO:0043066	Negative regulation of apoptotic process	25	455	4.13E−17
GO:0045893	Positive regulation of transcription, DNA-templated	24	515	8.12E−15
GO:0007165	Signal transduction	21	1161	6.17E−06
GO:0010628	Positive regulation of gene expression	18	262	9.90E−14
GO:0042493	Response to drug	17	304	1.37E−11
GO:0006915	Apoptotic process	17	567	1.08E−07
GO:0006954	Inflammatory response	16	379	3.26E−09
GO:0000122	Negative regulation of transcription from RNA polymerase II promoter	16	720	1.19E−05
GO:0008285	Negative regulation of cell proliferation	15	396	4.72E−08
GO:0032355	Response to estradiol	14	91	3.49E−15
GO:0008284	Positive regulation of cell proliferation	14	466	2.15E−06
GO:0071456	Cellular response to hypoxia	13	96	2.22E−13
GO:0001525	Angiogenesis	13	223	4.64E−09
GO:0006468	Protein phosphorylation	13	456	9.88E−06
GO:0045892	Negative regulation of transcription, DNA-templated	13	499	2.42E−05
GO:0001666	Response to hypoxia	12	172	3.51E−09
GO:0006974	Cellular response to DNA damage stimulus	12	208	2.54E−08
GO:0000165	MAPK cascade	12	262	2.66E−07
GO:0008283	Cell proliferation	12	366	6.90E−06

**Fig. 6:**
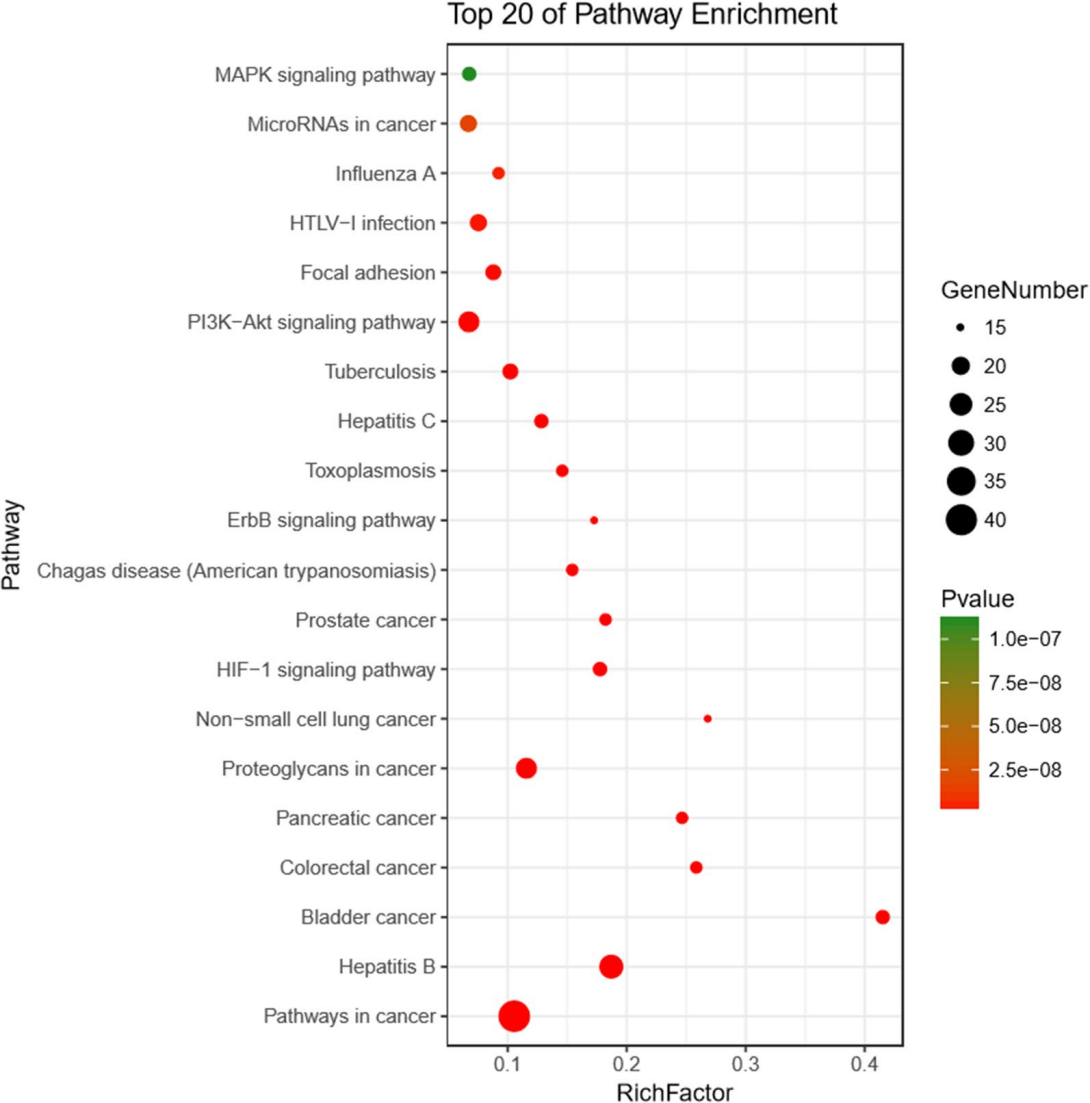
KEGG analysis for the major targets of YCHD

The KEGG pathway enrichment analysis was conducted using DAVID webserver. The target-pathway was built to delve into the mechanisms of potential targets acting on their corresponding signal pathways (Fig. 6; Table 4). YCHD was reported integrating multiple signaling pathways on cancers, immune system, infectious diseases, etc. Furthermore, YCHD probably exerted the therapeutic effects on cholangiocarcinoma by regulating signaling pathways, which included hepatitis B, the MAPK signaling pathway, the PI3K-Akt signaling pathway, and MicroRNAs in cancer.

**Table 4:** Main KEGG pathways significantly relating to major hubs

Term	Pathway	Count	Pop Hits	P Value
hsa05200	Pathways in cancer	41	393	6.63E−27
hsa05161	Hepatitis B	27	145	1.73E−23
hsa05205	Proteoglycans in cancer	23	200	3.70E−15
hsa04151	PI3K-Akt signaling pathway	23	345	2.43E−10
hsa05166	HTLV-I infection	19	254	2.58E−09
hsa05206	MicroRNAs in cancer	19	286	1.70E−08
hsa05152	Tuberculosis	18	177	6.66E−11
hsa04510	Focal adhesion	18	206	7.34E−10
hsa05219	Bladder cancer	17	41	1.18E−20
hsa04066	HIF-1 signaling pathway	17	96	4.41E−14
hsa05160	Hepatitis C	17	133	8.19E−12
hsa04010	MAPK signaling pathway	17	253	1.11E−07
hsa05210	Colorectal cancer	16	62	7.74E−16
hsa05212	Pancreatic cancer	16	65	1.78E−15
hsa05215	Prostate cancer	16	88	2.09E−13
hsa05142	Chagas disease (American trypanosomiasis)	16	104	2.67E−12
hsa05145	Toxoplasmosis	16	110	6.18E−12
hsa05164	Influenza A	16	174	4.54E−09
hsa05223	Non-small cell lung cancer	15	56	4.75E−15
hsa04012	ErbB signaling pathway	15	87	3.24E−12

### Herb‐compound target‐cholangiocarcinoma Network analysis

**Fig. 7:**
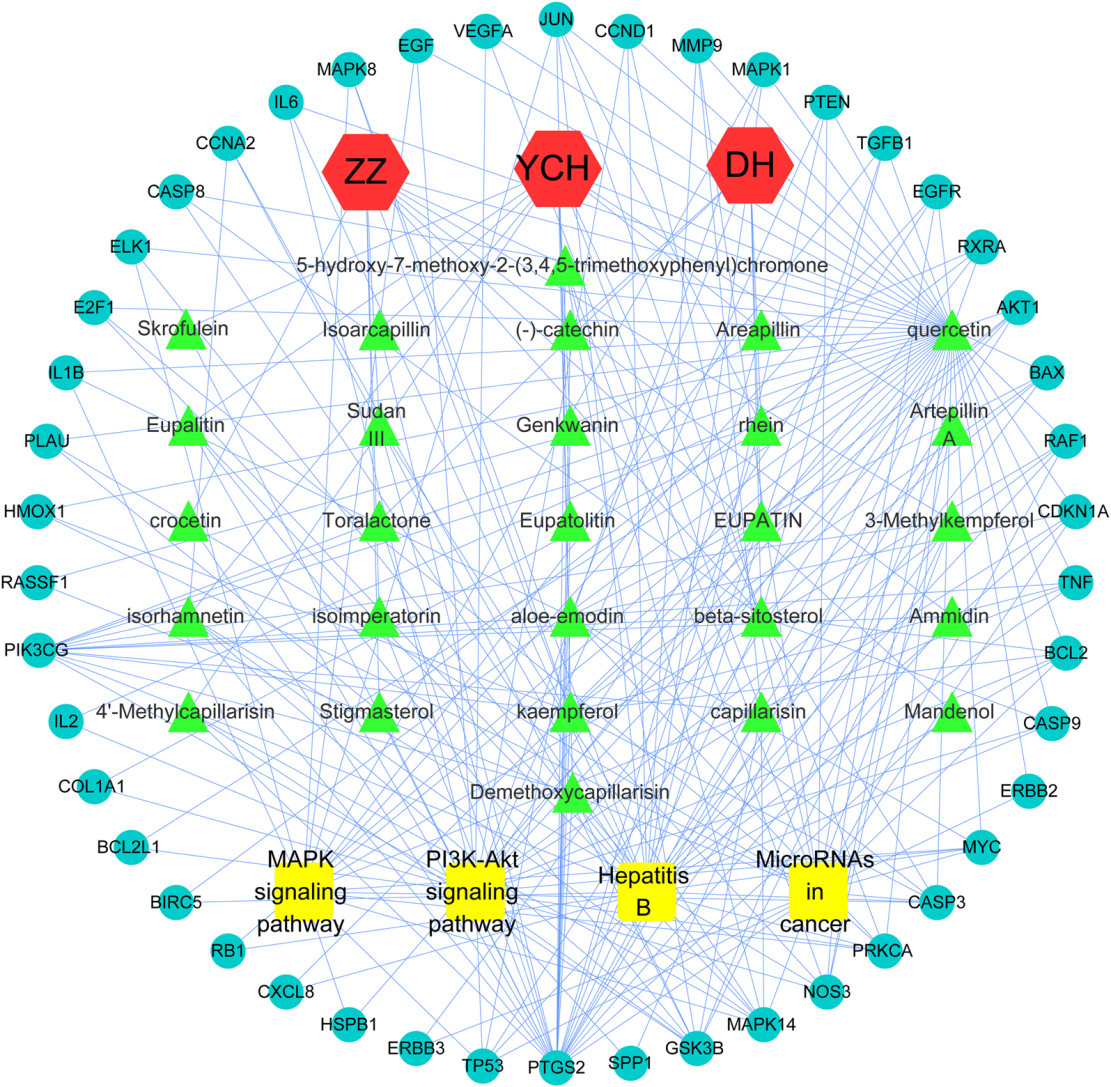
The herb-compound Target-cholangiocarcinoma network

To illustrate the relationship between three herbs of YCHD and their corresponding compound targets and cholangiocarcinoma targets, the herb-compound target-cholangiocarcinoma network was built. Based on this network, 80 nodes(3 herbs, 27 compounds, 46 targets, and 4 pathways) and 247 edges were identified (Fig. 7).

### Verification of compound-target interaction

**Fig. 8:**
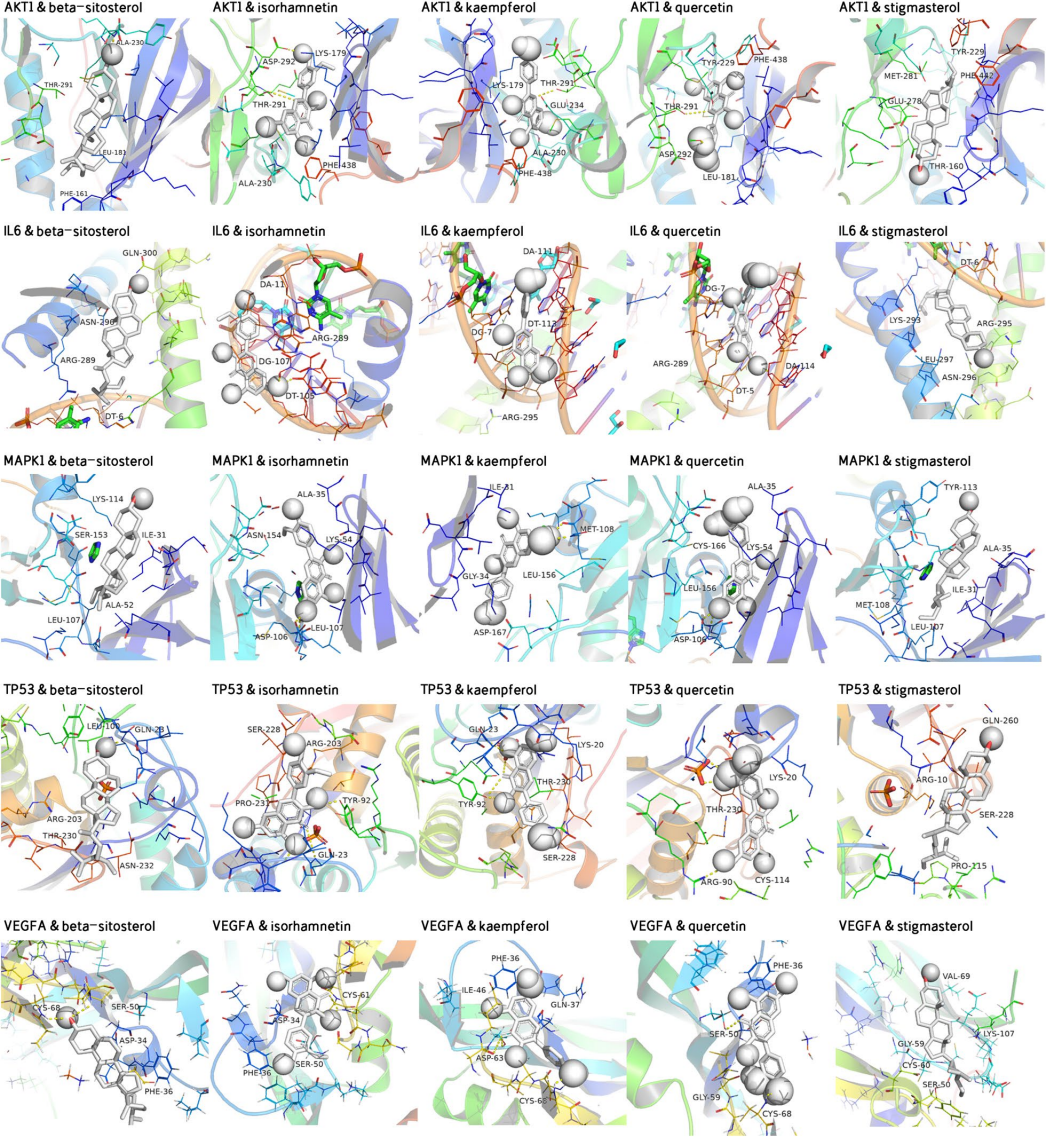
The conformations of main active compounds and major hub targets

The molecular docking was performed to further investigate interactions between vital active compounds and major targets. The binding affinity lower than − 5.0 kcal/mol indicates that the confirmations have good interactions [22]. In this study, molecular docking results showed that the conformations of active compounds and major protein targets showed good binding interactions, and the interactions were also reliable. The results of binding affinity was shown in Table 5. The conformations of key active compounds and major hub targets were displayed in Fig. 8.

**Table 5:** Virtual docking of five vital active compounds from YCHD for cholangiocarcinoma targets

Compound	Structure	Binding affinity/ (kcal/mol)				
		AKT1	IL6	MAPK1	TP53	VEGFA
Quercetin		− 7.8	− 8.1	− 7.4	− 7.7	− 7.1
Kaempferol		− 7.6	− 8.0	− 7.4	− 7.5	− 6.9
Beta-sitosterol		− 8.7	− 7.0	− 6.9	− 7.1	− 6.5
Isorhamnetin		− 7.7	− 8.0	− 7.2	− 7.7	− 7.6
Stigmasterol		− 7.9	− 7.0	− 6.7	− 7.7	− 6.6

*YCHD *Yinchenhao Decoction

### 3.7 YCHD inhibited the proliferation of cholangiocarcinoma cells

**Fig. 9:**
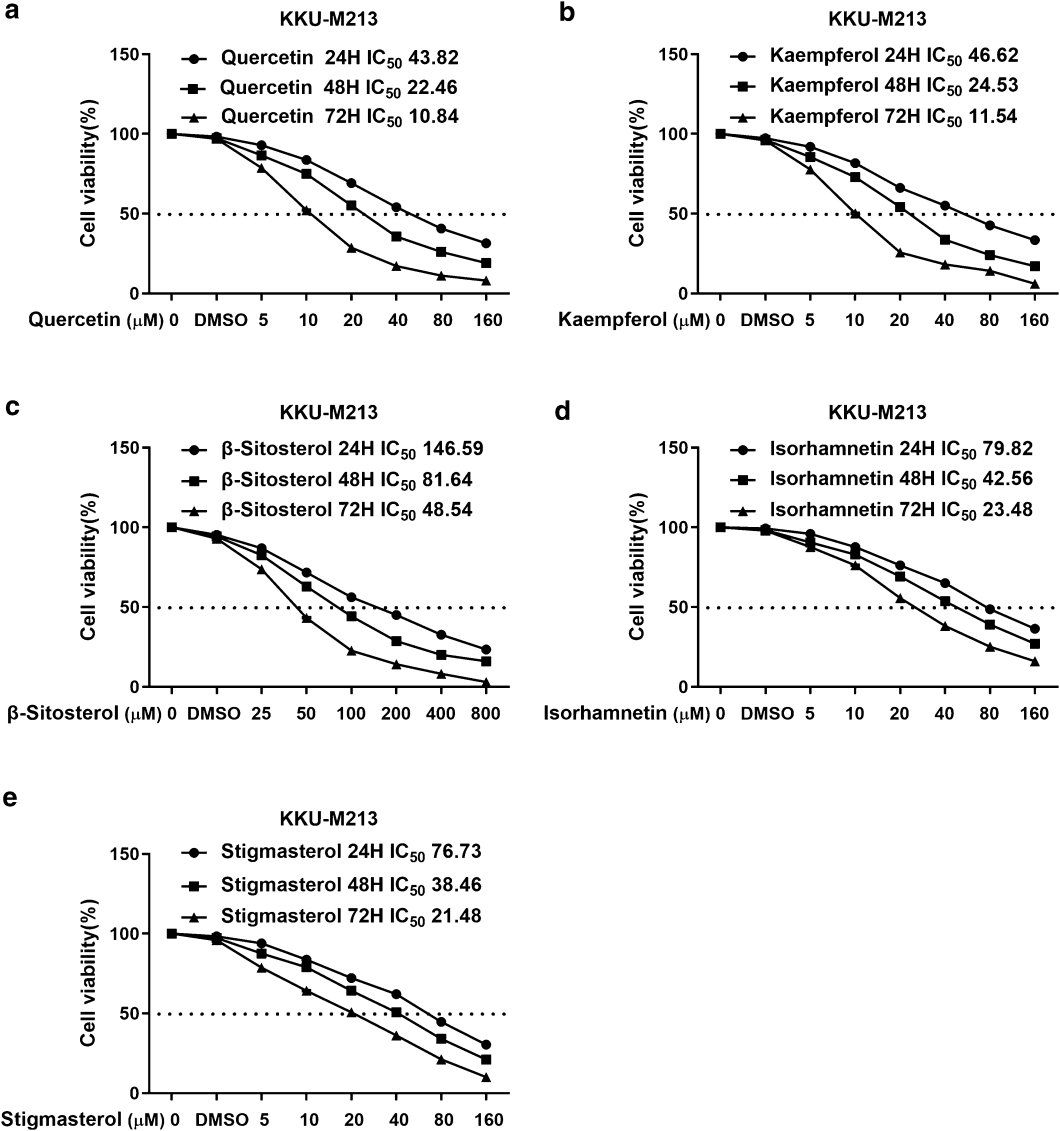
Proliferative inhibitory effects of five active compounds of YCHD treatment, including quercetin (**a**), kaempferol (**b**), beta-sitosterol (**c**), isorhamnetin (**d**), and stigmasterol (**e**) on KKU-M213 cells. Drug concentration-cell viability curves were generated based on the cell viability assay. All data were expressed as mean ± SD

Five active compounds including quercetin, kaempferol, beta-sitosterol, isorhamnetin, and stigmasterol were observed to inhibit cholangiocarcinoma cell proliferation in a concentration-dependent manner. The 72 h IC_50_ values of quercetin, kaempferol, beta-sitosterol, isorhamnetin, stigmasterol were 10.84, 11.54, 48.54, 42.56, and 23.48, respectively, revealing that these five active compounds for the KKU-M213 cell line showed a significant reduction in IC_50_ values over time. The cell viability curves were shown in Fig. 9.

### Quercetin and kaempferol induced apoptosis of cholangiocarcinoma cells

**Fig. 10:**
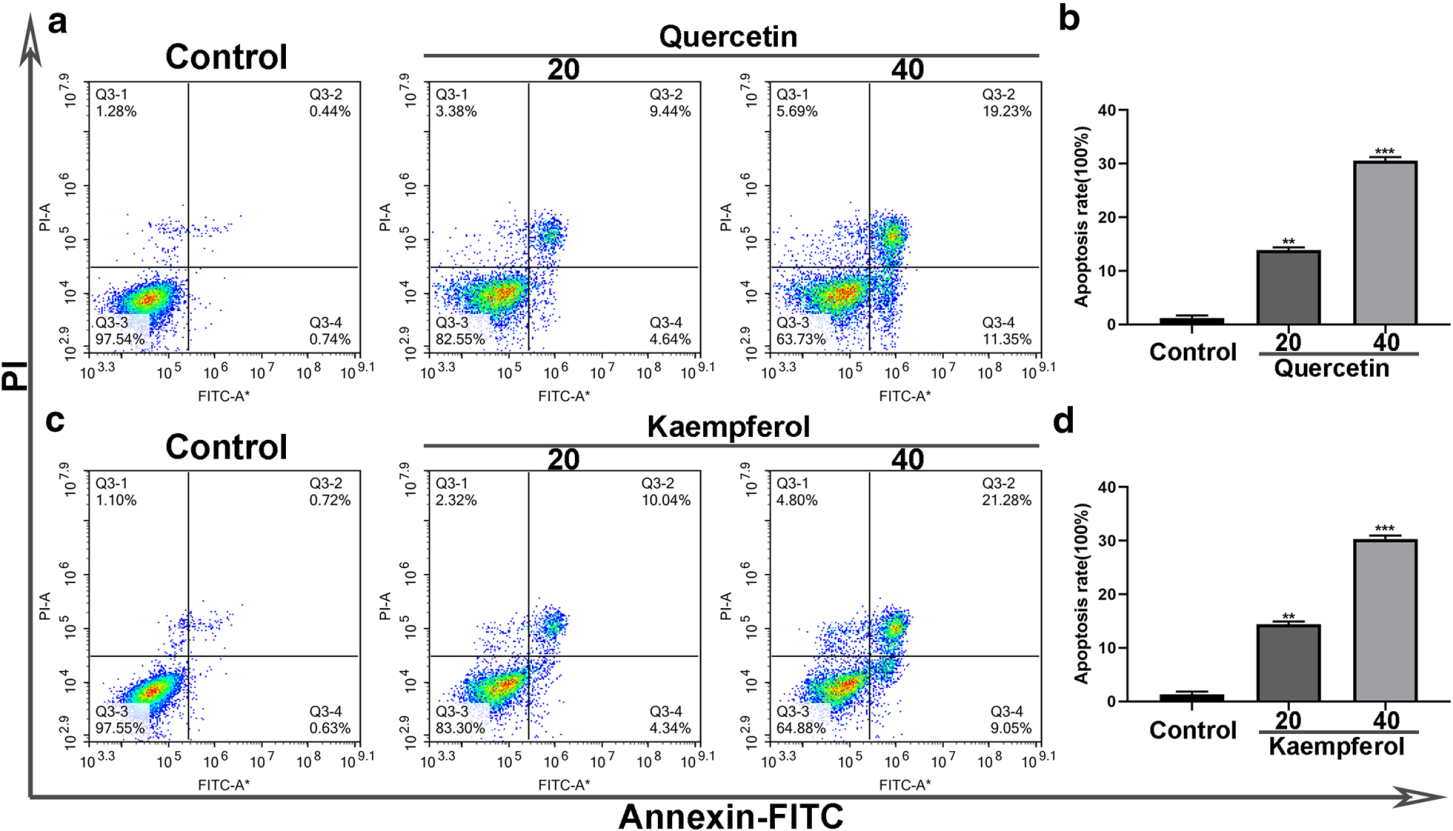
Representative profiles showing apoptosis of treated with quercetin and kaempferol alone. As determined by annexin V-fluorescein isothiocyanate (FITC) and propidium iodide (PI) staining, quercetin and kaempferol alone induced apoptosis of KKU-M213 cells (**a**, **c**). Data represented the cell population in cell cycle arrest of KKU-M213 cells (**b**, **d**). All data were expressed as mean ± SD (^**^P < 0.01; ^***^P < 0.001)

Higher doses of quercetin and kaempferol increased the apoptosis rate in PI-A cells compared with the control group, indicating that cholangiocarcinoma cells treated with quercetin and kaempferol may induce apoptosis (Fig. 10).

### Effect of quercetin and kaempferol on AKT, P53, MAPK and VEGFA mRNA exprssion levels in cholangiocarcinoma cells

**Fig. 11:**
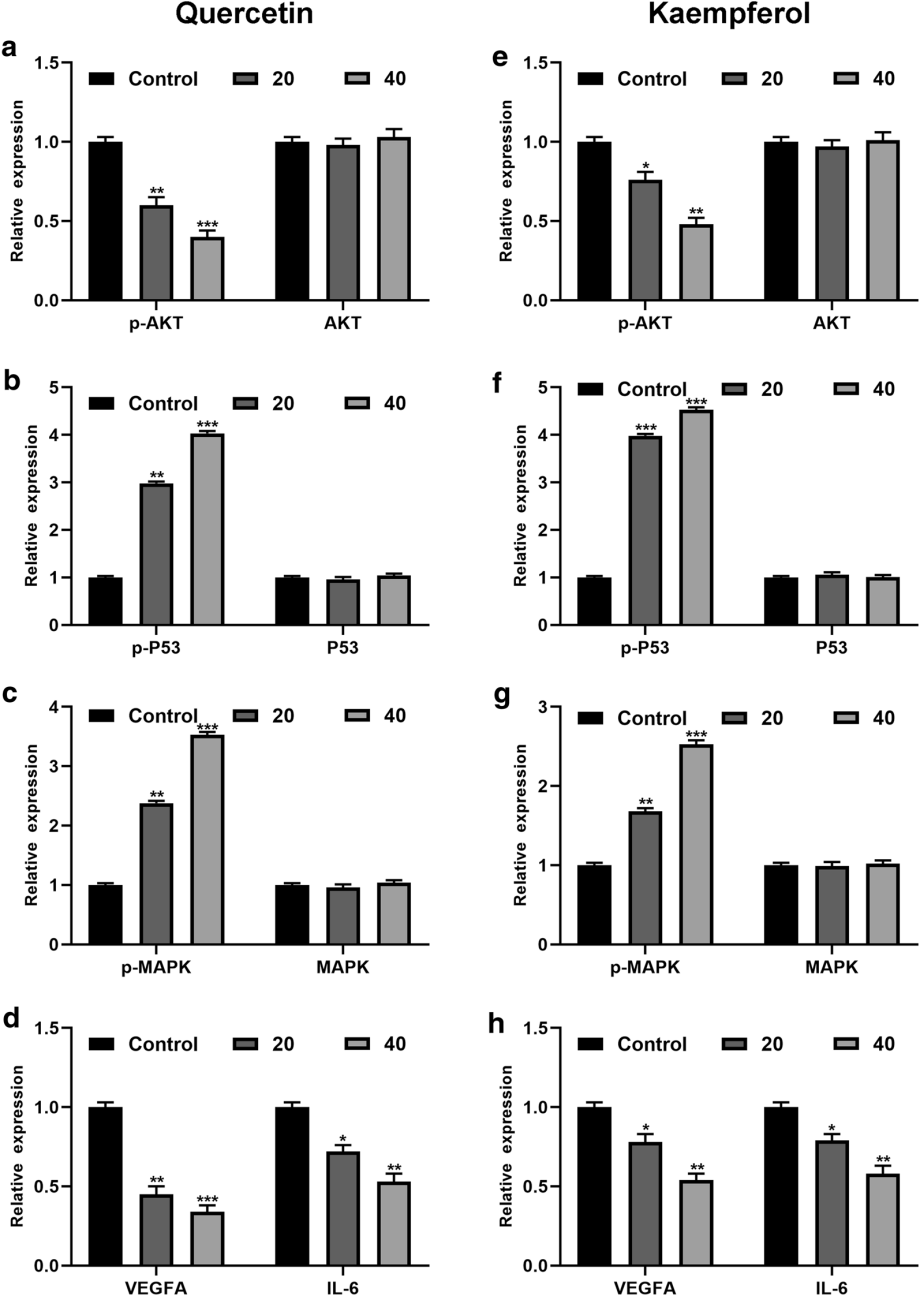
The expression of AKT, P53, MAPK and VEGFA mRNA levels were examined by real-time PCR treated with quercetin (**a**–**d**) and kaempferol (**e**–**h**)

Several key targets including AKT, P53, MAPK and VEGFA mRNA expression levels were validated by PCR. Quercetin and kaempferol were found to decrease levels of AKT and VEGFA, and increase P53 and MAPK levels. The results were depicted in Fig. 11.

### Quercetin combined with kaempferol regulated the protein expression of AKT, P53, MAPK, VEGFA and IL-6 in cell cycle control in cholangiocarcinoma cells

**Fig. 12:**
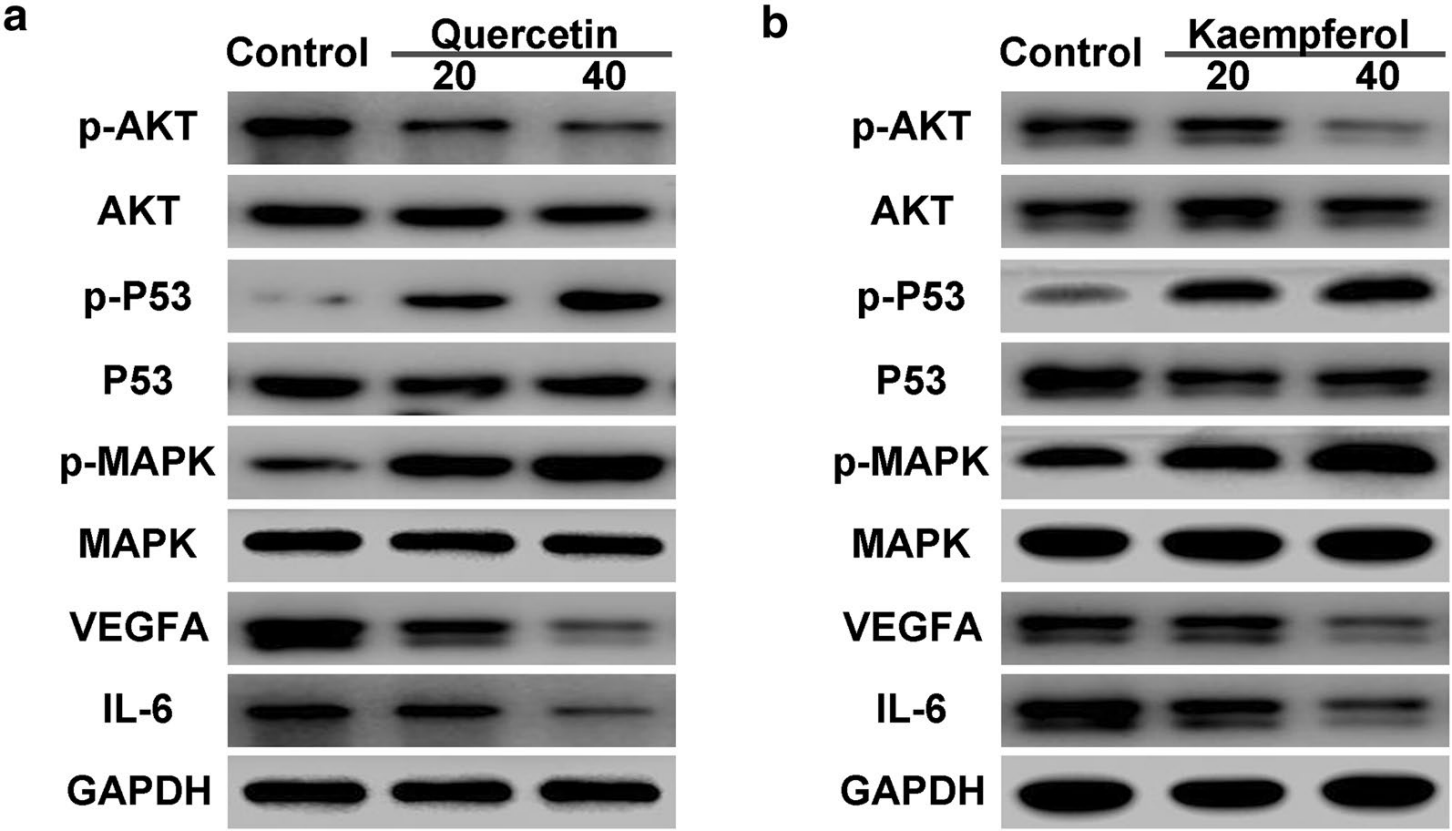
Protein expression levels of AKT, P53, MAPK, VEGFA, IL-6 and GAPDH in cell cycle control in KKU-M213 cells treated with quercetin (**a**) and kaempferol (**b**)

The targets including AKT, P53, MAPK, VEGFA and IL-6 were measured by western blot analysis. As shown in Fig. 12, quercetin and kaempferol decreased the expression of AKT, VEGFA and IL-6, and increased the expression of P53 and MAPK.

## Discussion

Cholangiocarcinoma refers to an epithelial cell malignancy with poor prognosis. Existing therapeutic strategies for patients with cholangiocarcinoma consist of surgery, liver transplantation and chemotherapy. Surgery is a prioritized treatment for all subtypes of cholangiocarcinoma, while lymph nodes and vascular structures should be considered. It was elucidated that for perihilar cholangiocarcinoma patients who have undergone either surgical treatment or liver transplantation, their 5-year survival rates were relatively low [1]. Besides, chemotherapy might cause adverse effects and lower patients’ quality of life [11, 23].

Accordingly, traditional Chinese medicine, an effective and safe complementary and alternative therapy, is considered to be able to treat cholangiocarcinoma. YCHD is a notable traditional Chinese medicine prescription, which consisting of three Chinese herbs, namely, YCH, ZZ and DH; it has been adopted to clear heat, eliminate dampness and remove jaundice [24]. As demonstrated by pharmacological researches, YCHD can modulate inflammatory as well as immune response, ameliorate liver function, and regulate multiple pathways in cancer [13, 25]. YCHD exerts therapeutic effects on liver disorders and metabolic diseases, whereas its mechanisms on cholangiocarcinoma remain unclear. In the present study, network pharmacology approach was adopted to describe the relationship among active compounds, compound targets and signaling pathways, and experimental methods were employed, thereby revealing the potential mechanisms of YCHD.

According to the results achieved here, 32 active compounds in YCHD with 209 compound targets were identified, suggesting that YCHD exerted its pharmacological effects on treating cholangiocarcinoma via multiple targets. Quercetin, kaempferol, beta-sitosterol, isorhamnetin, and stigmasterol were identified as the vital active compounds with top five degrees. As for quercetin, existing studies have indicated that quercetin is capable of inhibiting cell proliferation of cancer cell lines and regulating cancer metabolisms by modulating the PI3K-Akt-mTOR signaling and MAPK/ERK1/2 pathways [26, 27]. A previous experimental research has proved that quercetin could inhibit inflammatory and tumorigenesis processes and suppress growth and migration of cholangiocarcinoma cell lines [28]. Kaempferol is reported to inhibit the growth and metastasis of cholangiocarcinoma through suppressing the PI3K-AKT pathway and their downstream proteins [29]. As to beta-Sitosterol, it has anticancer properties associated with cell cycle, proliferation, apoptosis, etc. [30]. Isorhamnetin, a flavonoid metabolite, has been demonstrated to be able to produce anti-tumor effects by inhibiting the expression of NF-κB [31]. With regard to stigmasterol, it’s a major phytosterol in herbal plants, which can downregulate levels of inflammatory cytokines involving TNF-α,repress VEGF signaling, produce anti-angiogenic effects, thus inhibit cholangiocarcinoma growth in mice [32]. On the whole, it was speculated that YCHD is a multicomponent formula with multitarget therapeutic effects. Associations between these active compounds and cholangiocarcinoma are supposed to be deeply investigated.

In this study, AKT1, IL6, MAPK1, TP53,and VEGFA were identified as the 5 hub protein targets related to cholangiocarcinoma. Quercetin and kaempferol were proved to decrease the protein expression of AKT, VEGFA and IL-6, and increase P53 and MAPK levels.Previous study has reported that combined mTOR and AKT inhibition may significantly inhibit the tumor growth and proliferation of cholangiocarcinoma cell lines [33]. Similarly, the levels of AKT and IL6 can be decreased by genistein, which is associated with inhibitory effects on growth of cholangiocarcinoma cells [34]. Regarding MAPK1, it can be activated by osteopontin, a phosphorylatedglycoprotein involved in various human cancers, and implicated in activating the MEK/MAPK pathway, therefore promote the growth and metastasis of intrahepatic cholangiocarcinoma [35]. TP53 loss may drive the reprogramming of hepatocytes to biliary cells, which may be related to the formation of intrahepatic cholangiocarcinoma [36]. VEGFA plays an important part in tumor angiogenesis. An experimental research has verified that interactions of LOXL2 with GATA6 can induce the expression of VEGFA, which may promote angiogenesis in cholangiocarcinoma subcutaneous tumorsand tumor growth [37].

The GO analysis has demonstrated that YCHD is associated with the major biological process (e.g., positive regulation of transcription from RNA polymerase II promoter, negative regulation of apoptosis process, and positive regulation of transcription, DNA-templated). Regarding the RNA polymerase II promoter, a transcriptional regulatory element, was served as a sequence initiating transcription and regulating epigenetics [38, 39]. The RNA polymerase II binding to the tumor suppressor CDC73 contributed to transcriptional repression of oncogenes, conversely, oncogene overexpression could down-regulate CDC73 in tumors and lead to cell proliferation [40]. As for apoptosis, a physiological cell death, can regulate development of organisms, remove cells no longer available or differentiated ones incompatible with the body and maintain organizational homeostasis [41]. Tumor will be induced once cell apoptosis is out of control. Caused by many risk factors, chronicinflammation and cholestasis are driving forces in cholangiocarcinoma development [42]. The accumulation of bile acids from cholestasis lead to pH reduction, increase in apoptosis and activation of ERK1/2,Akt and NF-κB pathways [43]. A previous research [44] has confirmed that cholangiocarcinoma cell apoptosis was induced by THZ1 inhibiting the synthesis of antiapoptotic protein, and THZ1 could down-regulate the transcriprion of RNA polymerase II promoter in cancer cells and produce anti-tumour effects. In addition, the transcription of DNA-templated possibly related to the anti-tumor property of THZ1 [44]. The existing studies haven’t reported the relationship between DNA-templated transcription and cholangiocarcinoma, and this may become the further research direction. Therefore, we could speculate that the effects of YCHD on cholangiocarcinoma may relate to the above-mentioned biological processes.

As revealed from the KEGG pathway analysis, YCHD produced therapeutic effects on cholangiocarcinoma by regulating pathways (e.g., hepatitis B, the MAPK signaling pathway, the PI3K-Akt signaling pathway, and MicroRNAs in cancer). Recent epidemiological studies reported hepatitis B virus infection as a risk factor of intrahepatic cholangiocarcinoma [45, 46]. Intrahepatic cholangiocarcinoma patients with current and past hepatitis B virus infection were reported to display a better prognosis as compared with those without a history of hepatitis B virus infection [47].

Experimental results in this study confirmed that the active compounds of YCHD inhibited cell proliferation, induced apoptosis of cholangiocarcinoma cells, thus delayed the progression of cholangiocarcinoma. The MAPK signaling pathway participated in a variety of biological processes such as inflammatory response, cell differentiation, cell apoptosis and tumor invasiveness and so on [48]. A previous study confirmed that the MAPK signaling pathway was activated in intrahepatic cholangiocarcinoma cells by TRIM44, a protein involved in several kinds of cancers, to inhibit cell apoptosis and promote tumour invasion and metastasis [49]. For PI3K-Akt signaling pathway, it has been proved to regulate cell growth and proliferation and be critical to cancers [50, 51]. As one of the most intensively explored signaling pathways in tumorigenesis, PI3K-Akt signaling pathway may participate in the initiation, maintenance and metastasis of cholangiocarcinoma [52]. Existing research has elucidated that theinhibitory of transcription factor 21 mediated by PI3K-Akt signaling pathway could inhibit the progression of cholangiocarcinoma [53]. With respect to microRNAs, a group of small RNAs regulating genes expression, are modulators to suppress or progress tumor [54]. According to one previous research, microRNA-329 had an inhibitory effect on the expression of PTTG1 and inactivated the MAPK signaling pathway to inhibit the cholangiocarcinoma cell proliferation, induce cell cycle arrest as well as promote cell apoptosis, thus prevented the progression of cholangiocarcinoma [55]. Accordingly, it could be assumed that YCHD regulated hepatitis B, the MAPK signaling pathway, the PI3K-Akt signaling pathway, as well as MicroRNAs in cancer, so it could treat cholangiocarcinoma.

The molecular docking validated that vital active compounds and major targets showed good binding interactions. Except for regulation of quercetin on the PI3K-AKT and MAPK pathways described above, quercetin has the ability of inhibiting cell migration and angiogenesis mediated by downregulating VEGFA levels in glioblastoma cells [56], whereas no study has focused on the relationship of quercetin and VEGFA in treating cholangiocarcinoma. Kaempferol can treat cholangiocarcinoma through the PI3K-AKT pathway [29]. Moreover,kaempferol lowers the levels of TP53, which is involved in attenuating apoptosis mediated by Cisplatin [57]. In terms of beta-sitosterol, it is reported to induce MAPK phosphorylation, downregulate the PI3K/Akt,thus promote cell apoptosis and cell death [58]. Results from KEGG pathway analysis indicated that the action mechanisms of YCHD for cholangiocarcinoma were implicated in hub targets of AKT1 and MAPK1. We could speculate that YCHD exerts therapeutic effects on cholangiocarcinoma through these active compounds, target genes and signaling pathways.

Most of the recent studies have applied the network pharmacology approach to screen active compounds, describe interactions between active compounds and relevant targets, and predict action mechanisms of various diseases [59, 60]. In addition to the network pharmacology methods, this study employed experimental methods to further explore the predictive mechanisms of YCHD for cholangiocarcinoma at molecular and cellular levels. However, there are some limitations in this study. First, some critical targets and active compounds may be ignored due to the incomplete information of databases. Second, multiple signaling pathways of YCHD acting on cholangiocarcinoma were predicted using the network pharmacology strategy, but the contribution of each pathway hasn’t been detected. Third, our current experiment and network pharmacology results provide directions for subsequent researches, whereas mechanisms still need fully investigated in future researches.

##  Conclusions

In this study, quercetin, kaempferol, beta-sitosterol, isorhamnetin, and stigmasterol were identified as the vital active compounds, and AKT1, IL6, MAPK1, TP53 as well as VEGFA were considered as the major targets. The molecular docking revealed that these active compounds and major targets showed good binding interactions. YCHD may treat cholangiocarcinoma via signaling pathways including hepatitis B, the MAPK signaling pathway, the PI3K-Akt signaling pathway, and MicroRNAs in cancer. Experimental researches provided evidence that YCHD showed therapeutic effects on cholangiocarcinoma by regulating related target protein, inhibiting cell proliferation, and increasing cell apoptosis rate. This study demonstrated potential pharmacological mechanisms of YCHD acting on cholangiocarcinoma; it can be referenced for clinical application of YCHD.

## Data Availability

The datasets used and/or analysed during the current study are available from the corresponding author on reasonable request.

## Abbreviations

YCHD: Yinchenhao Decoction
TCMSP: Traditional Chinese Medicine System Pharmacology Database and Analysis Platform
DAVID: Database for Annotation, Visualization, and Integrated Discovery
GO: Gene ontology
KEGG: Kyoto Encyclopedia of Genes and Genomes
OB: Oral bioavailability
DL: Drug-likeness
PDB: Protein Data Bank

## References

[1] Razumilava N, Gregory J (2014). Gores cholangiocarcinoma. Lancet.

[2] Yao D, Kunam V, Li X (2014). A review of the clinical diagnosis and therapy of cholangiocarcinoma. J Int Med Res.

[3] Patel T (2002). Worldwide trends in mortality from biliary tract malignancies. BMC Cancer.

[4] Razumilava N, Gores GJ (2014). Cholangiocarcinoma. Lancet.

[5] Palmer WC, Patel T (2012). Are common factors involved in the pathogenesis of primary liver cancers? A meta-analysis of risk factors for intrahepatic cholangiocarcinoma. J Hepatol.

[6] Charbel H, Al-Kawas F (2011). Cholangiocarcinoma: epidemiology, risk factors, pathogenesis, and diagnosis. Curr Gastroenterol Rep.

[7] Ruzzenente A, Conci S, Valdegamberi A (2015). Role of surgery in the treatment of intrahepatic cholangiocarcinoma. Eur Rev Med Pharmacol Sci.

[8] Huang J, Jiang B, Yang Y (2018). Influencing factors for the prognosis of patients with early-stage intrahepatic cholangiocarcinoma after radical resection. J Clin Hepatol.

[9] Lim K, Han S, Oh D (2012). Outcome of infusional 5-fluorouracil, doxorubicin, and mitomycin-C (iFAM) chemotherapy and analysis of prognostic factors in patients with refractory advanced biliary tract cancer. Oncology.

[10] Valle J, Wasan H, Palmer D (2010). Cisplatin plus gemcitabine versus gemcitabine for biliary tract cancer. N Engl J Med.

[11] Liu S, Zhou L, An L (2019). Implementation of comprehensive rehabilitation therapy in postoperative care of patients with cholangiocarcinoma and its impact on patients’ quality of life. Exp Ther Med.

[12] Chen Z, Ma X, Zhao Y (2015). Yinchenhao decoction in the treatment of cholestasis: a systematic review and meta-analysis. J Ethnopharmacol.

[13] Jiang S, Hu X, Liu P (2015). Immunomodulation and liver protection of Yinchenhao decoction against concanavalin A-induced chronic liver injury in mice. J Integr Med.

[14] Qi J (2018). Clinical Observation on Modified Yinchenhao Decoction Combined with Tegafur Gimeracil Oteracil Potassium Capsule in the Treatment of Advanced Pancreatic Cancer. Guangming J Chin Med.

[15] Song Z, Song H, Song P (2018). Clinical observation of modified Yinchenhao tang combined with cisplatin Intraperi-toneal perfusion for Ascites Hepatoma. J New Chin Med.

[16] Li S, Zhang B, Jiang D (2010). Herb network construction and co-module analysis for uncovering the combination rule of traditional Chinese herbal formulae. BMC Bioinformatics.

[17] Ru J, Li P, Wang J (2014). TCMSP: a database of systems pharmacology for drug discovery from herbal medicines. J Cheminformatics.

[18] Huang L, Xie D, Yu Y (2018). TCMID 2.0: a comprehensive resource for TCM. Nucleic Acids Res.

[19] Kramer C, Podewitz M, Ertl P (2015). Unique macrocycles in the Taiwan traditional Chinese medicine database. Planta Med.

[20] Chao W, Lin B (2011). Bioactivities of major constituents isolated from Angelica sinensis (Danggui). Chin Med.

[21] Tao W, Xu X, Wang X (2013). Network pharmacology-based prediction of the active ingredients and potential targets of Chinese herbal Radix Curcumae formula for application to cardiovascular disease. J Ethnopharmacol.

[22] Li X, Xu X, Wang J (2012). A system-level investigation into the mechanisms of Chinese Traditional Medicine: compound danshen formula for cardiovascular disease treatment. PLoS One.

[23] He F, Wang M, Li K (2018). Efficacy analysis of hepatic arterial infusion in combination with intravenous gemcitabine chemotherapy for advanced intrahepatic cholangiocarcinoma. Chin J General Surg.

[24] Guo Y, Li J, Mao T (2017). Effect of Combined Prescription of Linggui Zhugan Tang and Yinchenhao Tang on Nrf2/ARE Signaling Pathway in Rats with Non-alcoholic Steatohepatitis. Chin J Exp Trad Med Formul.

[25] Huang J, Cheung F, Tan H (2017). Identification of the active compounds and significant pathways of yinchenhao decoction based on network pharmacology. Mol Med Rep.

[26] Reyes-Farias M, Carrasco-Pozo C (2019). The anti-cancer effect of Quercetin: molecular implications in cancer metabolism. Int J Mol Sci.

[27] Erdogan S, Turkekul K, Dibirdik I (2018). Midkine downregulation increases the efficacy of quercetin on prostate cancer stem cell survival and migration through PI3K/AKT and MAPK/ERK pathway. Biomed Pharmacother.

[28] Senggunprai L, Kukongviriyapan V, Prawan A (2014). Quercetin and EGCG exhibit chemopreventive effects in cholangiocarcinoma cells via suppression of JAK/STAT signaling pathway. Phytother Res.

[29] Qin Y, Cui W, Yang X (2016). Kaempferol inhibits the growth and metastasis of cholangiocarcinoma in vitro and in vivo. Acta Biochim Biophys Sin.

[30] Bin Sayeed MS, Ameen SS, Beta-Sitosterol (2015). A Promising but Orphan Nutraceutical to Fight Against Cancer. Nutr Cancer.

[31] Manu KA, Shanmugam MK, Ramachandran L (2015). Isorhamnetin augments the anti-tumor effect of capecitabine through the negative regulation of NF-κB signaling cascade in gastric cancer. Cancer Lett.

[32] Kangsamaksin T, Chaithongyot S, Wootthichairangsan C (2017). Lupeol and stigmasterol suppress tumor angiogenesis and inhibit cholangiocarcinoma growth in mice via downregulation of tumor necrosis factor-α. PLoS One.

[33] Ewald F, Grabinski N, Grottke A (2013). Combined targeting of AKT and mTOR using MK-2206 and RAD001 is synergistic in the treatment of cholangiocarcinoma. Int J Cancer.

[34] Tanjak P, Thiantanawat A, Watcharasit P (2018). Genistein reduces the activation of AKT and EGFR, and the production of IL6 in cholangiocarcinoma cells involving estrogen and estrogen receptors. Int J Oncol.

[35] Zheng Y, Zhou C, Yu XX (2018). Osteopontin promotes metastasis of intrahepatic cholangiocarcinoma through recruiting MAPK1 and mediating Ser675 phosphorylation of β-Catenin. Cell Death Dis.

[36] Hill MA, Alexander WB, Guo B (2018). Kras and Tp53 mutations cause cholangiocyte- and hepatocyte-derived cholangiocarcinoma. Cancer Res.

[37] Peng T, Deng X, Tian F (2019). The interaction of LOXL2 with GATA6 induces VEGFA expression and angiogenesis in cholangiocarcinoma. Int J Oncol.

[38] Kadonaga J (2012). Perspectives on the RNA polymerase II core promoter. Wiley Interdiscip Rev Dev Biol.

[39] Chujan S, Suriyo T, Satayavivad J (2019). Integrative in silico and in vitro transcriptomics analysis revealed gene expression changes and oncogenic features of normal cholangiocytes after chronic alcohol exposure. Int J Mol Sci.

[40] Rather M, Swamy S, Gopinath K (2014). Transcriptional repression of tumor suppressor CDC73, encoding an RNA polymerase II interactor, by Wilms tumor 1 protein (WT1) promotes cell proliferation: implication for cancer therapeutics. J Biol Chem.

[41] Celli A, Que F (1998). Dysregulation of apoptosis in the cholangiopathies and cholangiocarcinoma. Semin Liver Dis.

[42] Andersen JB (2015). Molecular pathogenesis of intrahepatic cholangiocarcinoma. J Hepatobiliary Pancreat Sci.

[43] Labib PL, Goodchild G, Pereira SP (2019). Molecular pathogenesis of cholangiocarcinoma. BMC Cancer.

[44] Huang T, Ding X, Xu G (2019). CDK7 inhibitor THZ1 inhibits MCL1 synthesis and drives cholangiocarcinoma apoptosis in combination with BCL2/BCL-XL inhibitor ABT-263. Cell Death Dis.

[45] Zhang H, Yang T, Wu M (2016). Intrahepatic cholangiocarcinoma: epidemiology, risk factors, diagnosis and surgical management. Cancer Lett.

[46] Jeong S, Tong Y, Sha M (2017). Hepatitis B virus-associated intrahepatic cholangiocarcinoma: a malignancy of distinctive characteristics between hepatocellular carcinoma and intrahepatic cholangiocarcinoma. Oncotarget.

[47] Jiang B, Ge R, Sun L (2011). Clinical parameters predicting survival duration after hepatectomy for intrahepatic cholangiocarcinoma. Can J Gastroenterol.

[48] Chen C, Nelson L, Ávila MA (2019). Mitogen-Activated Protein Kinases (MAPKs) and cholangiocarcinoma: the missing link. Cells.

[49] Peng R, Zhang PF, Zhang C (2018). Elevated TRIM44 promotes intrahepatic cholangiocarcinoma progression by inducing cell EMT via MAPK signaling. Cancer Med..

[50] Li H, Hu J, Wu S (2016). Auranofin-mediated inhibition of PI3K/AKT/mTOR axis and anticancer activity in non-small cell lung cancer cells. Oncotarget.

[51] Burris HA (2013). Overcoming acquired resistance to anticancer therapy: focus on the PI3K/AKT/mTOR pathway. Cancer Chemother Pharmacol.

[52] Polivka J, Janku F (2014). Molecular targets for cancer therapy in the PI3K/AKT/mTOR pathway. Pharmacol Ther.

[53] Duan H, Li B, Zhaung X (2019). TCF21 inhibits tumor-associated angiogenesis and suppresses the growth of cholangiocarcinoma by targeting PI3K/Akt and ERK signaling. Am J PhysiolGastrointestLiver Physiol.

[54] Chang Y, Yin F, Fan GF (2017). Downregulation of miR-329-3p is associated with worse prognosis in patients withcervical cancer. Eur Rev Med Pharmacol Sci.

[55] Hu Z, Zheng C, Su H (2019). MicroRNA-329-mediated PTTG1 downregulation inactivates the MAPK signaling pathway to suppress cell proliferation and tumor growth in cholangiocarcinoma. J Cell Biochem.

[56] Liu Y, Tang ZG, Yang JQ (2017). Low concentration of quercetin antagonizes the invasion and angiogenesis of human glioblastoma U251 cells. Onco Targets Ther.

[57] Wang Z, Sun W, Sun X (2020). Kaempferol ameliorates Cisplatin induced nephrotoxicity by modulating oxidative stress, inflammation and apoptosis via ERK and NF-κB pathways. AMB Express.

[58] Moon D, Lee K, Choi Y (2007). Beta-sitosterol-induced-apoptosis is mediated by the activation of ERK and the downregulation of Akt in MCA-102 murine fibrosarcoma cells. Int Immunopharmacol.

[59] Yu G, Zhang Y, Ren W (2016). Network pharmacology-based identification of key pharmacological pathways of Yin-Huang-Qing-Fei capsule acting on chronic bronchitis. Int J Chron Obstruct Pulmon Dis.

[60] Lee W, Lee C, Kim Y (2019). The methodological trends of traditional herbal medicine employing network pharmacology. Biomolecules.

